# Somatic development in children with Shwachman-Diamond syndrome

**DOI:** 10.1186/s13052-020-00919-z

**Published:** 2020-10-12

**Authors:** Agnieszka Bogusz-Wójcik, Honorata Kołodziejczyk, Maja Klaudel-Dreszler, Grzegorz Oracz, Joanna Pawłowska, Mieczysław Szalecki

**Affiliations:** 1 Department of Endocrinology and Diabetology, The Children’s Memorial Health Institute, Av. Dzieci Polskich 20, 04-736 Warsaw, Poland; 2 Anthropology Department, The Children’s Memorial Health Institute, Warsaw, Poland; 3 Department of Gastroenterology, Hepatology, Feeding Disorders and Paediatrics, The Children’s Memorial Health Institute, Warsaw, Poland; 4 Collegium Medicum, University of Jan Kochanowski, Kielce, Poland

**Keywords:** Somatic development, Shwachman-Diamond syndrome, Anthropometric parameters, Short stature

## Abstract

**Background:**

Shwachman-Diamond syndrome (SDS) is a rare genetic, multi-systemic disease characterized by exocrine pancreatic insufficiency, immune deficiency, bone marrow failure and skeletal abnormalities. Most patients present with failure in somatic development and short stature, but systematic data concerning those features are limited. The aim of the study was to assess the prevalence of failure in somatic development in the children with SDS.

**Methods:**

An analysis of anthropometric measurements of 21 patients (14 girls and 7 boys),aged 2 to 17 years (mean age 6.3 years) with SDS diagnosed in The Children’s Memorial Health Institute in Warsaw, Poland was performed. The patients were measured using a Holtain Limited stadiometer, an electronic scale, a Harpenden anthropometer, a metric tape and a spreading caliper. The assessed anthropometric parameters were expressed as standard deviation scores in relation to the reference values in Poland, suitable for sex as well as calendar and growth age.

**Results:**

A total of 66 measurements was collected and analyzed with a median number of 3 observations per patient. The group of boys presented with a significantly lower height (− 3.0 SD, *p* < 0.0001) and BMI (− 1.4 SD, *p* < 0.00001), and in the relation to the growth age a lower weight (− 1.0 SD, *p* < 0.001) as well as a smaller chest width (− 0.9 SD, *p* < 0.05), hip width (− 0,5 SD, p < 0,05) and lower limb length (− 0,5 SD, p < 0,05). The group of girls also showed significantly lower height (− 2.6 SD, *p* < 0.00001) and BMI (− 0.8 SD, p < 0.00001), and in relation to the growth age, lower weight (− 0.5 SD, *p* < 0.001) as well as decreased width of the chest (− 1.7 SD, *p* < 0.0001) and shoulder (− 1.0 SD, p < 0.001) were observed. Boys and girls were also characterized by significantly decreased circumference and width of head, additionally, girls had also smaller head length.

**Conclusions:**

Patients with SDS have abnormal somatic development. Both boys and girls are characterized by short stature, decreased weight, BMI, leg length, chest width as well as circumference and width of head. Anthropometric measurements provide important data on the process of growth and body proportions in children with SDS.

## Introduction

Shwachman-Diamond syndrome (SDS) is a rare autosomal recessive disorder first described in 1964 [1]. It is characterized by pancreatic exocrine insufficiency, immune deficiency, bone marrow failure, and skeletal abnormalities [2]. In addition, the liver, kidneys, teeth and brain may also be affected [3]. SDS is also associated in about 15% with increased risk of developing myelodysplastic syndrome (MDS) and acute myeloid leukemia (AML) [4]. In addition, failure in somatic development and short stature are frequently observed in patients with SDS as well.

Pancreatic insufficiency arises early in infancy and is characterized by replacement of exocrine components with fatty tissue, but at the same time, islets of Langerhans and ductal architecture are preserved. Pancreatic function spontaneously improves overtime in almost 50% of patients [5].

Most patients exhibit persistent or intermittent neutropenia responsible for recurrent and often severe bacterial infections [2].

In 2002, the *SBDS* gene was described as being involved in the syndrome in question, and identified on chromosome 7q1 1[6]. Nevertheless, it has recently been reported that other genes, including *DNAJC21*, *EFL1* and *SRP54* are also associated with a SDS-like phenotype [7–9]. All genes associated with SDS are involved in ribosome biogenesis, strengthening the postulate that SDS is a ribosomopathy [10].

Approximately 10% of patients with clinical symptoms of SDS do not have mutations in the *SBDS* gene [6, 11]. However, the negative result of genetic evaluation should not exclude the diagnosis. A precise clinical evaluation is significant to diagnose the presence of the syndrome [2].

Several clinical studies reported that growth failure is mainly due to inadequate nutrient intake in the presence or in the absence of feeding difficulties, pancreatic insufficiency and recurrent infections. After diagnosis and the start of an appropriate therapy, growth rate is restored to normal level in most of the children with SDS, even though it consistently remains below the third percentile for height and weight [5].

There is a significant variability in clinical phenotype, even within families. Most studies on the somatic development of children with SDS have been limited to weight and height, with only a few studies assessing BMI [5, 12–16]. The majority of studies demonstrated that SDS patients present with growth failure, nevertheless systematic data concerning those features are limited. Therefore we have decided to examine development of children with SDS more closely.

Accordingly, we prospectively analyzed a large group of SDS patients, and collected all available anthropometric measurements.

The aim of the study was to assess the prevalence of failure in somatic development in children with SDS and compare failure to thrive in boys and girls.

## Patients and methods

### Patients

Overall, 21 patients (14 girls and 7 boys) with Shwachman-Diamond syndrome diagnosed and hospitalized in Children’s Memorial Health Institute, Warsaw, Poland between 2005 and 2017 were recruited. Patients were observed during follow-up lasting at least 1 year. The average calendar age of girls was 6.2 ± 4.3 years (95% CI: 4.8–7.6), and boys were 6.4 ± 4.4 years (95% CI: 4.5–8.4). The study was consistent with the Helsinki declaration and received IRB approval (33/KBE/2018). Written parental consent was obtained in all cases. The SDS diagnosis was based on the clinical criteria proposed by Dror et al. in 2011^2^ and confirmed by molecular diagnostics in 19 (90%) out of 21 patients (MEDGEN, Poland). Two patients without mutation in *SBDS* gene did not agree for further genetic analyses, but present with all clinical criteria. Growth hormone (GH) deficiency was observed in 6 patients requiring GH treatment, but only measurements carried out before GH substitution therapy were analyzed. All patients with height < − 2 SD below mean were evaluated for GH deficiency. GH deficiency was diagnosed if stimulated peak of GH on 2 standard stimulation tests (with clonidine, glucagon and/or arginine) were < 10 ng/mL and if insulin-like growth factor 1 (IGF-1) and insulin-like growth factor binding protein 3 (IGF-BP3) were low or below the normal range for age. At 2 years of age, 2 patients underwent haematopoetic stem cell transplantation. Their measurements were collected at age 5 and 6. Pancreatic insufficiency was defined as fecal fat quantities of > 5 g/day 72-h-fecal fat quantification using the modified van de Kamer method remains the gold standard for diagnosing EPI with fat maldigestion. For fecal fat quantification using modified van de Kamer method samples were pooled over 3-day period while consuming normal patient’s diet. Enzyme replacement therapy, if implemented, was withheld 7 days before stool collection. In patients who had more than one test, the highest result was taken into consideration [17].

### Anthropometric parameters

Axiological parameters including body high, weight and BMI (w/h^2^; w = weight/kilogram, h = hight/meter) were collected in all 21 patients following standard protocol. 18 patients (86%) underwent selected anthropometric body and head measurements, using a standard measuring techniquein their underwearonly [18, 19]. Measurements were performed at the Department of Anthropology at Children’s Memorial Health Institute. Body height (B-v) was measured using a Holtain Limited stadiometer with an accuracy of 0.1 cm. Body weight was assessed using an electronic scale with an accuracy of 0.1 kg. The upper limb length (a-da), i.e. the distance from the shoulder appendage to the tip of the third finger pad, the lower limb length (B-sy), i.e. the distance from the upper edge of the pubic symphysis to the base, and the trunk length (sst-sy), i.e. the distance from the jugular notchof the sternum in the median sagittal plane to the edge of the pubic symphysis, were measured with an accuracy of 0.1 cm using a Harpenden anthropometer. The upper and lower limb length were measured only for right side of the body. In clinical examination no asymmetry in length of body limbs were found. Dimensions of shoulder width (aa), i.e. the place most laterally and upward on the outer edge of the shoulder appendix, the chest width (thl-thl), i.e. points in the axillary midline lateral at the height of the nipples, the hips width (ic-ic) at the most lateral position on the iliac crest and the chest depth (xi-ths) at the point located on the junction of the sternum stem with the xiphoid process in the median plane were measured using a spreading caliper with an accuracy of 0.1 cm [18, 19]. The head circumference was measured at the largest place, i.e. the frontal tumors and protuberance occipital with the metric tape with an accuracy of 0.5 cm, while the head length (g-op) was measured in the median plane by glabella points and opisthocranion, finally the head width (eu-eu) was measured between euryon side points. Both measurements were made with a spreading caliper with an accuracy of 0.1 cm [18, 19]. Detailed data on the analysed anthropometric features are given in Table 1.The body and head dimensions obtained from anthropometric measurements were compared with the references of the Mother and Child Institute, Warsaw, Poland [20]. Data were standardized and referred to the average values of the population of healthy Polish children, expressed as standard deviation score (SD) separately for girls and boys, according to the formula: standard deviation score = (X examined - X population) / SD of the population. All anthropometric features were assessed in relation to both calendar and growth age (Tables 3, 4 and 5). The calendar age is the time elapsed from birth to the day of the examination, while through the growth age, we determine the actual level of advancement in growth and maturing. The growth age is equal to the calendar age if the height corresponds to 50 centiles. In clinical practice, in all patients whose height is not on 50 centiles, values of some anthropometric measurements, such as body weight, length and width dimensions, should refer to the actual height, i.e. growth age. It differs in the case of head measurements. Its values should be related to the calendar age of patients. This is because the head grows differently than the rest of the body.

**Table 1:** Characteristics of anthropometric parameters of boys and girls with Shwachman-Diamond syndrome (mean ± standard error; 95% confidence interval)

Somatic characteristic	Boys *n* = 7		Girls *n* = 14	
	x ± SE	95% Cl	x ± SE	95% CI
Height (cm)	105.5 ± 6.3	92.3–118.7	103.1 ± 4.1	94.8–111.4
Weight (kg)	17.7 ± 2.1	13.5–22.1	17.8 ± 1.7	14.3–21.4
Body Mass Index (BMI) (kg/m^2^)	14.3 ± 0.2	13.9–14.8	15.3 ± 0.4	14.5–16.0
Upper limb length (cm)	44.0 ± 3.4	36.5–51.6	42.7 ± 2.4	37.8–47.7
Lower limb length (cm)	48.2 ± 4.0	39.3–57.1	45.3 ± 2.8	39.6–51.1
Trunk length (cm)	32.4 ± 1.9	28.0–36.7	32.4 ± 1.4	29.5–35.2
Shoulder width (cm)	22.9 ± 1.4	19.8–26.1	21.8 ± 1.0	19.8–23.9
Chest width (cm)	16.4 ± 0.7	14.8–18.1	15.4 ± 0.6	14.2–16.6
Chest depth (cm)	12.3 ± 0.6	11.5–13.1	12.1 ± 0.4	11.2–12.9
Hip width (cm)	16.2 ± 0.9	14.2–18.2	16.5 ± 0.7	14.9–18.1
Head circumference (cm)	47.4 ± 0.6	46.2–48.6	47.7 ± 0.7	46.3–49.1
Head length (mm)	164.6 ± 2.9	158.0–171.1	159.5 ± 3.7	151.6–167.5
Head width (mm)	130 ± 1.2	127.6–133.1	131.4 ± 2.1	126.9–136.0

### Statistical methods

Statistical analysis was performed using Statistica 7.0. (StatSoft, INC). The results obtained are presented as the mean value with a standard error or standard deviation and a 95% confidence interval. Shapiro-Wilk test was used for the comparison of the normality of variable distributions. Student’s t-test was used to assess anthropometric parameters. A two-sided test of differences between two means was used to calculate the difference between the average of a given patient’s characteristics and the average of the population. The local significance level is set to 0.05, that is, *P* values of 0.05 were considered statistically noticeable.

## Results

Twenty-one (14 girls/7 boys) patients diagnosed with SDS, were recruited to our study. A total of 62 measurements were collected from 2005 to 2019, with a median number of 3 observations per patient. All patients were of Caucasian and Polish origin with the median age at diagnosis of 2.7 years (range: 0.1–12.5 years)**.** The median gestational age was 39 weeks (range: 33–41 weeks), the median weight at birth was − 1.2 SD (ranging from − 1.9 to + 0.7 SD) in the group of boys and − 0.7 SD (ranging from − 1.7 to + 1.5 SD) with regard to the girls. The median length at birth was − 1.4 SD (ranging from − 1.7 to − 0.6 SD) for the boys and − 0.95 SD (ranging from − 1.7 to + 0.1 SD) for the girls.

Pancreatic insufficiency was observed in 6 boys (86%) and 11 girls (79%). Nine patients (43%) were on pancreatic enzyme replacement therapy during observation period.

All the patients, except for one, presented with neutropenia. Two patients (10%) at the age of 2 years old, underwent haematopoetic stem cell transplantation, due to bone marrow failure. Skeletal abnormalities such as metaphyseal dysostosis, cupping of the anterior ribs, delayed bone age, osteoporosis, clinodactylies and thoracic dystrophy were found in the analyzed group of patients. Thoracic dystrophy led to a preliminary diagnosis of Jeune syndrome or asphyxiating thoracic dystrophy (ATD) in one patient. GH deficiency requiring GH substitution therapy was diagnosed in 6 cases (29%) and treatment was started at a mean 8.3 years of age (range: 5–11 years). Five patients are still receiving growth hormone treatment with good treatment response after 2 years of therapy while one patient received the treatment around puberty with moderate response. Other endocrinology diseases such as hypothyroidism (1 patient), primary adrenal insufficiency (1 patient) and type 1 diabetes (1 patient) were also diagnosed in this cohort. The patients’ characteristics are shown in Table 2.

**Table 2:** Patients characteristics

Patients’ characteristics	Study cohort	
	Male	Female
Total	7	14
Gestational age, weeks	39 [38–40]	39 [33–41]
Preterm	0	2 (14)
Weight at birth, SD	− 1.2 [− 1.9–0.7]	− 0.7 [− 1.7–1.5]
Length at birth, SD	−1.4 [− 1.7– − 0.6]	−0.95 [− 1.7–0.1]
Age at diagnosis, years	1.4 [0.1–3.5]	3.3 [0.1–12.5]
Pancreatic insufficiency	6 (86)	11 (79)
Neutropenia	7 (100)	13 (93)
Heart problems	0	1 (7)
Skeletal abnormalities	7 (100)	14 (100)
Hematopoietic stem cell transplantation	0	2 (14)
GH treatment	2 (29)	4 (29)

*SD* standard deviation score, *GH* growth hormone

Values are n, n (%)or median [range]

The percentage is given in brackets and refers to the total count of male and female patients

In the analysed group of boys, the average calendar age was 6.4 ± 4.4 years (range: 0.8–13.8 years) and the average growth age was 4.9 ± 4 years (range: 0.2–11.7 years). In the girls’ group, the average calendar age was 6.2 ± 4.3 years (range: 0.3–17.7 years) and the average growth age was 4.5 ± 3.3 years (range: 0.1–12.2 years). Significant differences were observed between the calendar and growth age in both groups, which the average of about 1.5 years. Both groups were characterised by significantly lower body height (boys − 3.0 SD girls − 2.6 SD) and lower BMI (boys − 1.4 SD, girls − 0.8 SD) with regard to the calendar age, and significantly lower weight (boys − 1.0 SD, girls − 0.5 SD) with regard to the growth age in relation to peer population (Table 3).

**Table 3:** Differences in somatic characteristics (SDS) concerning thenutritional statusof boys and girls with Shwachman-Diamond syndrome (mean ± standard deviation)

Somatic characteristic	Boys *n*= 7		Girls *N* = 14	
	Calendar age 6.4 ± 4.4	Growth age 4.9 ± 4	Calendar age 6.2 ± 4.3	Growth age 4.5 ± 3.3
	standard deviation score			
Height	−3.0 ± 2.0***	0	−2.6 ± 1.6****	0
Weight	−2.3 ± 1.3***	−1.0 ± 0.9**	−1.8 ± 1.0****	−0.5 ± 0.9**
Body Mass Index (BMI)	−1.4 ± 0.8****	−1.3 ± 0.9****	−0.8 ± 1.0****	−0.7 ± 1.3*

*p < 0,05; **p < 0,005; ***p < 0,0001; ****p < 0,00001; 0 – no data; parameters calculated only to calendar age regarding anthropological standards

As far as the growth age is concerned (Table 4), the examined boys showed significantly decreased dimensions of lower limb length (− 0.5 SD), chest width (− 0.9 SD) and hip width (− 0.5 SD), as well as longer upper limbs (+ 0.3 SD) and trunk (+ 0.2 SD). With regard to the remaining features (Table 5), i.e. shoulder width and chest depth, no significant differences were found in relation to the population of healthy children. Furthermore, as regards the calendar age, significant differences were observed in head parameters such as circumference (− 2.5 SD) and width (− 2 SD) (Table 5).

**Table 4:** Differences in somatic characteristics (SDS) concerning body proportions of boys and girls with Shwachman-Diamond syndrome (mean ± standard deviation)

Somatic characteristic	Boys *n* = 6		Girls *N* = 12	
	Calendar age	Growth age	Calendar age	Growth age
	standard deviation score			
Upper limb length	−1.45 ± 1.1**	0.3 ± 0.3*	−1.9 ± 1.1****	0.2 ± 0.6
Lower limb length	−2.7 ± 1.2***	−0.5 ± 0.6*	−3.5 ± 1.7***	−0.7 ± 0.7***
Trunk length	−1.0 ± 0.8**	0.2 ± 0.8*	−0.7 ± 1.1**	0.9 ± 0.8***
Shoulder width	−1.7 ± 1.8**	−0.23 ± 1.7	−2.8 ± 1.6***	−1.0 ± 1.2**
Chest width	− 1.96 ± 1.5**	− 0.9 ± 1.2*	− 2.7 ± 1.5****	− 1.7 ± 1.2***
Chest depth	−0.7 ± 1.1*	−0.1 ± 0.9	−0.9 ± 1.0***	−0.2 ± 1.3
Hip width	−1.8 ± 0.8****	−0.5 ± 0.5*	−1.6 ± 1.4***	0.1 ± 1.2

*p < 0,05; **p < 0,005; ***p < 0,0001; ****p < 0,00001

**Table 5:** Differences in anthropometric head parameters (SDS) of boys and girls with Shwachman-Diamond syndrome (mean ± standard deviation)

Somatic characteristic	Boys *n* = 6		Girls *N* = 12	
	Calendar age	Growth age	Calendar age	Growth age
	standard deviation score			
Head circumference	−2.5 ± 1.1****	2.8 ± 18.4	−2.2 ± 1.4****	−0.9 ± 1.1***
Head length	− 0.6 ± 1.0	0	−0.9 ± 1.3*	0
Head width	−2.0 ± 0.8****	0	−1.4 ± 0.9****	0

*p < 0,05; **p < 0,005; ***p < 0,0001; ****p < 0,00001; 0 – no data; parameters calculated only to calendar age regarding anthropological standards

The analyses of the data concerning the group of the girls, it was noticed that in relation to the growth age (Tables 4 and 5) they were characterized by a significantly lower length of the lower limbs (− 0.7 SD), shoulder width (− 1.0 SD), chest width (− 1.7 SD), and increased dimensions of trunk length (+ 0.8 SD). As far as the remaining examined features are concerned, in relation to the growth age: longer upper limbs (+ 0.2 SD), as well as wider hips (+ 0.1 SD) and decreased chest depth (− 0.3 SD) were found. Additionally, significant differences were also observed in head parameters such as circumference (− 2.2 SD), length (− 0.9 SD) and width (− 1.4 SD) with regard to the calendar age (Table 5).

It is worth emphasising that both boys and girls, in relation to the growth age (Table 4), were characterized by longer trunk length (0.2 SD boys and 0.8 SD girls) and upper limb length (0.3 SD boys and 0.2 SD girls), as well as shorter lower limb length (− 0.5 SD boys and − 0.7 SD girls). Furthermore, with regard to the calendar age, both groups had significantly decreased head parameters such as head circumference and width (Table 5). The examined boys were shorter than girls and had lower weight and BMI (Table 3).

Based on the assessment of anthropometric parameters, it was found that the differences occurring in the studied boys and girls were visible both in relation to the calendar and growth age.

## Discussion

SDS is a rare multisystemic genetic disease with no well-defined prevalence, which is estimated between 1/76,563 and 1/200,000 [21]. Somatic development abnormalities and severe growth retardation are its characteristic features.

Typical skeletal changes are present in all patients with SDS, however their severity and location change with age [22]. In the cohort being the subject of analyses, all the patients had skeletal changes, but only 8 presented such abnormalities in the physical examination. The characteristic changes included: delayed appearance of secondary ossification centers, metaphyseal dysostosis and generalized osteopenia [22]. All the patients in the cohort who underwent bone mineral density measurements showed osteopenia. According to Rosendahl et al. and Toiviainen-Salo et al., who studied bone mineral density, bone biopsies and vitamin D and K status in SDS patients, there is a primary defect of bone metabolism in children affected with SDS [23, 24].

Until now, the description of the development of patients with SDS has been limited to the analysis of height, weight and BMI. In 2018, Cipolli et al. published the first growth charts for SDS patients from the Italian cohort of 0–8 years old [5]. This retrospective study includes 106 patients. The authors created specific charts for height, weight and BMI for SDS children which serve as helpful tools used in monitoring treatment efficiency and for the purposes of routine medical follow-up. The 50th percentile of SDS charts for weight and height is positioned on the 3rd percentile of regular charts, both for boys and girls [5].

However, other anthropometric parameters have not been well characterized so far. As to our best knowledge, this study is the first analysis of anthropometric measurements of body length and width dimensions, as well as head parameters, which allowed for a detailed analysis of the somatic development of SDS patients. It shows that patients affected with SDS need a proper and necessary assessment of physical development, referring to both the calendar and growth age. Assessing individual features only in relation to the calendar age, one can draw conclusions inadequately. As far as the development of healthy children is concerned, the pubertal growth spurt is a critical phase in children presenting SDS, with growth velocity significantly affecting their final height. The delayed bone age assessed in SDS patients [25] improves the prediction of their final height. It is worth emphasize, that children with SDS, as regards the growth age, had normal length of trunk and upper limbs indicating that metaphyseal dysostosis is mainly presented in lower limbs and ribs.

Analyzing BMI, height and weight harmonic trend on SDS growth charts, according to Cipolli et al., the growth retardation is influenced by the genetic defect rather than malabsorption/malnutrition or inherited factors [5]. Our analyses showed that weight and BMI of SDS patients with regard to the growth age, were not below − 2 SDS confirming that malnutrition is not the main factor leading to growth retardation. Children with poor growth can benefit from an endocrinological evaluation and tests for growth hormone deficiency [14].

Although short stature and failure to thrive are not included in diagnostic criteria of SDS, most papers [2, 5, 12–16] and our data indicate that abnormal somatic development comprises a cardinal symptom of the syndrome. It is common for most patients. Moreover, even in the absence of the other diagnostic criteria of SDS, it may become an indication for molecular diagnostics towards SDS. Anthropometric measures in presence of other clinical aspects suggestive of SDS, but in absence of classical mutations in SBDS gene, are important data to push us towards deeper genetic investigations (SBDS gene sequencing, other genes, etc.) to arrive at a diagnosis.

## Conclusion

In conclusion, our results confirm that short stature and abnormal somatic development are associated with this rare disease. Our study is the first assessment of anthropometric measurements of patients with SDS. We suggest the graphing height and weight referring to growth age to be routinely done in children and adolescents every 6–12 months to timely diagnosed growth failure. BMI should also be monitored.

## Data Availability

The datasets used and/or analysed during the current study are available from the corresponding author on request.

## Abbreviations

SDS: Shwachman-Diamond syndrome
SD: Standard deviation score
GH: Growth hormone
MDS: Myelodysplastic syndrome
AML: Acute myeloid leukemia
IGF-1: Insulin-like growth factor 1
IGF-BP3: Insulin-like growth factor binding protein 3
